# First Evidence of Dehydroabietic Acid Production by a Marine Phototrophic Gammaproteobacterium, the Purple Sulfur Bacterium *Allochromatium vinosum* MT86

**DOI:** 10.3390/md16080270

**Published:** 2018-08-04

**Authors:** Johannes F. Imhoff, Mingshuang Sun, Jutta Wiese, Marcus Tank, Axel Zeeck

**Affiliations:** 1 Department of Marine Microbiology, GEOMAR Helmholtz Centre for Ocean Research Kiel, 24105 Kiel, Germany; sunmin_wolf@163.com (M.S.); jwiese@geomar.de (J.W.); 2 Shenzhen Key Lab of Marine Genomics, BGI Fisheries, BGI, Building No.11, Beishan Industrial Zon, Yantian District, Shenzhen 518083, China; 3 Department of Biological Sciences, Tokyo Metropolitan University, Hachioji-shi, Tokyo 192-0397, Japan; mtank@tmu.ac.jp; 4 Bioviotica Naturstoffe GmbH, 37127 Dransfeld, Germany; zeeck@bioviotica.de

**Keywords:** anoxygenic phototrophic bacteria (APB), *Allochromatium vinosum*, secondary metabolites, dehydroabietic acid, antibiotic

## Abstract

The production of secondary metabolites by a new isolate of the purple sulfur bacterium *Allochromatium vinosum*, which had shown antibiotic activities during a preliminary study, revealed the production of several metabolites. Growth conditions suitable for the production of one of the compounds shown in the metabolite profile were established and compound **1** was purified. The molecular formula of compound **1** (C_20_H_28_O_2_) was determined by high resolution mass spectra, and its chemical structure by means of spectroscopic methods. The evaluation of these data revealed that the structure of the compound was identical to dehydroabietic acid, a compound known to be characteristically produced by conifer trees, but so far not known from bacteria, except cyanobacteria. The purified substance showed weak antibiotic activities against *Bacillus subtilis* and *Staphylococcus lentus* with IC_50_ values of 70.5 µM (±2.9) and 57.0 µM (±3.3), respectively.

## 1. Introduction

In contrast to the oxygenic cyanobacteria, little knowledge exists on secondary metabolites of the anoxygenic phototrophic bacteria. From a total of 200 isolates, only two identified as *Marichromatium purpuratum* showed antibiotic activity with a broad antimicrobial spectrum [1]. The activity was associated with the chromatophore fraction of the cells. Production of antibiotic substances has also been demonstrated in aerobic marine phototrophic bacteria affiliated with the genus *Roseobacter* and the compound produced during the whole growth cycle was identified as the new substance tropodithietic acid [2]. In addition, genes of PKS and NRPS biosynthetic pathways were identified in several isolates belonging to the *Roseobacter* clade [3].

In another study, three cytokinins were found in the cells of *Rhodospirillum rubrum,* one of these identified as zeatinriboside [4]. In *Rhodobacter sphaeroides*, two indole terpenoids were identified, both of which exhibited phytohormone activity and were regarded as results of biotransformation reactions of supplied substrates: rhodethrin was produced after growth with L-tryptophan [5] and rhodestrin was produced when grown on anthranilate [6]. In the same strain, a third indole ester was identified as an indole carboxylate esterified with a terpenoid alcohol after growth with 2-aminobenzoate of the same strain [7].

Though diterpenes have been extensively studied in plants and fungi, increasing evidence is accumulating that terpene biosynthesis is widespread also in bacteria [8,9,10]. In anoxygenic phototrophic bacteria, evidence exists for their presence in different phylogenetic branches. In *Chloroflexus aurantiacus*, e.g., verrucosan-2ß-ol [11] and in *Rhodospirillum rubrum* isoagathenediol [12] were identified.

The diterpenoids dehydroabietic and abietic acids are commonly associated with plants and fungi and are quite characteristically produced by conifer trees. They also have been identified recently in a wide range of cyanobacteria [13]. These authors suggested biosynthesis via the methylerythrol pathway (the only known terpene biosynthesis pathway in cyanobacteria) using pyruvate and glyceraldehyde-3-phosphate as substrates. They also conclude that the biosynthesis of these two compounds in plants has cyanobacterial origin because the synthesis of abietadiene takes place in the chloroplasts of the plants.

In the present study, initiated to identify secondary metabolites of phototrophic purple bacteria, which are Gammaproteobacteria, we have for the first time demonstrated the production of dehydroabietic acid by the purple sulfur bacterium *Allochromatium vinosum* MT86.

## 2. Results

### 2.1. Phylogenetic Analysis and Identification of the Isolate

Strain MT86 was isolated from a marine sandy rock pool near Trivendrum on the southwest coast of India and classified according to phenotypic properties and 16S rRNA gene sequence analysis as a strain of the species *Allochromatium vinosum.* Sequence identity with the type strain of *Allochromatium vinosum* DSM 180^T^ was 100% (Genbank accession number FM178268). It was found that crude cultures of strain MT86 revealed antibiotic activities and therefore it was selected for a more detailed study on the secondary metabolite production.

### 2.2. Metabolite Profiles, Fractionation of Extracts and Purification of Compound ***1***

**Figure 1:**
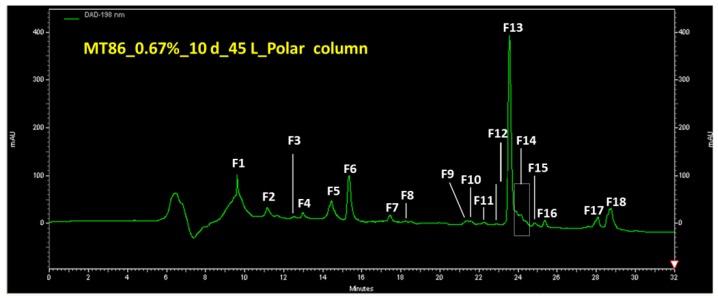
Semi-preparative HPLC (polar column) of an extract obtained from 45 L culture broth of MT86 with 0.7% salt. Peak F13 corresponds to compound **1**.

Metabolite profiles of culture extracts were compared after growth in Pfennigs medium using analytical HPLC-DAD/MS chromatography with two types of columns. Among a number of variations of the Pfennigs medium, a medium containing MgCl_2_ and supplemented with thiosulfate and acetate provided the best conditions for growth and metabolite production of *Allochromatium vinosum* strain MT86. The largest number of metabolites was produced under these conditions. A typical HPLC chromatogram of an extract from a batch culture grown in this medium is shown in Figure 1.

The compound with the peak identification number F13 and later identified as dehydroabietic acid was produced by *Allochromatium vinosum* MT86 under various growth conditions. It was excreted by the cells and detected in the culture supernatant, but not within the cells. By comparing various cultivation conditions, the best conditions for the production of dehydroabietic acid were determined to be in Pfennigs medium supplemented with 0.7% NaCl after incubation for 10 days in light.

Extracts of the culture supernatant were fractionated by semi-preparative HPLC by using the UV absorption to fractionate the peaks manually. The polar column (4 µL Polar-RP 80A, 250 mm × 21.20 mm, Phenomenex Synergi) was better suited for fractionation compared to the C18 column.

### 2.3. Purification of Compound ***1***

Compound **1** was prepared and purified from several culture batches with a total volume of 50 L and the purified fractions were combined. Purity of the substance from different batches was confirmed by HPLC-DAD/MS analysis and ^1^H-NMR. From these data, the identity of fractions from different batches was also confirmed. The purified fractions from different culture batches were pooled and purified. The final purification of the pooled batches was made on a Sephadex LH20 column with methanol as solvent. The pure substance (a final yield of 2.5 mg pure substance was obtained from culture supernatant of approximately 50 L) was used for chemical structure analysis (^1^H-NMR in CD3OD).

Purity check of compound **1** was made by separation by HPLC (column: Nucleodur C18, 5 um, 100 A, 250 mm × 3 mm; solvent: acetonitrile/water gradient with 0.1% TFA). Detection was at 220 nm and by ELSD. Compound **1** has a retention time of 22.27 min and appears to be rather lipophilic. The UV spectrum has a maximum at 215 nm and weak bands at 267/273 nm, which could derive from a phenyl ring.

### 2.4. Structure Analysis of Compound ***1***

**Figure 2:**
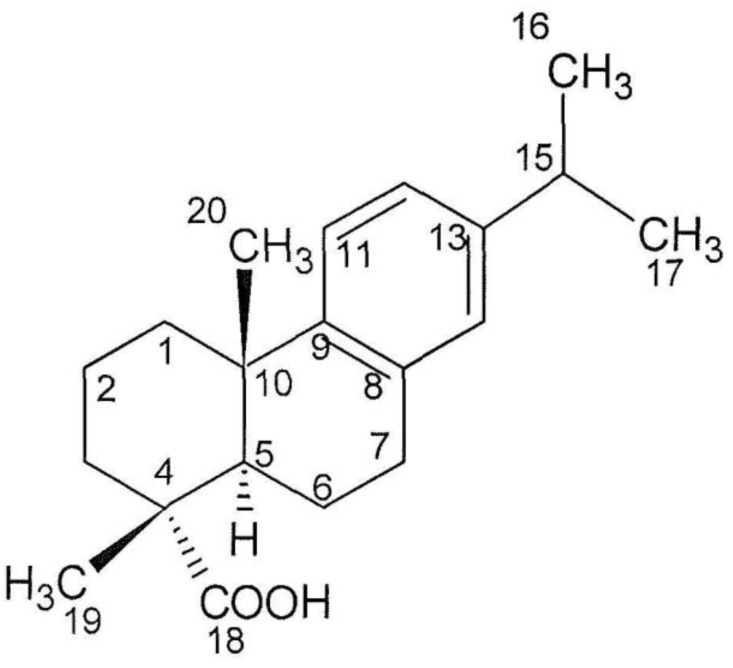
Structure of compound **1** (dehydroabietic acid).

**Figure 3:**
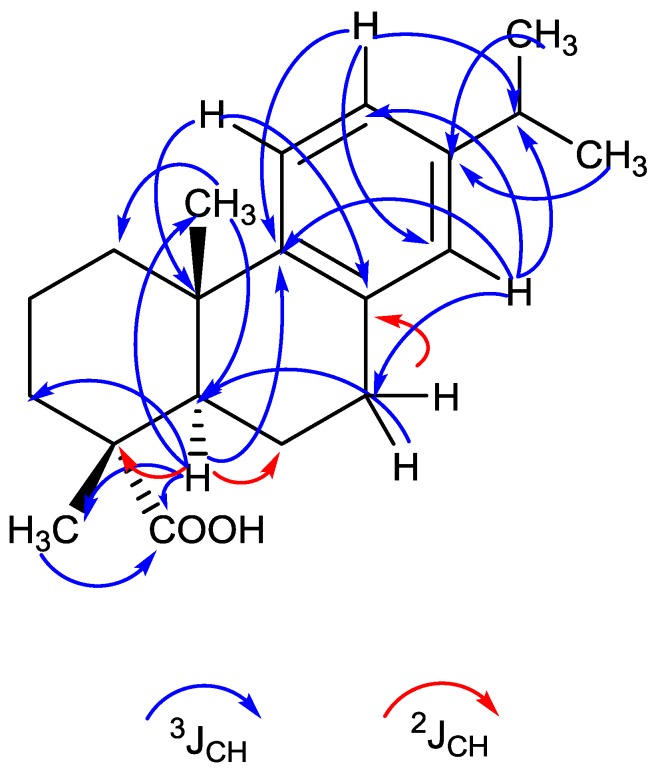
Molecular representation of the HMBC correlation of dehydroabietic acid through 3J and 2J coupling.

From the 12 apparent absorption peaks of the IR spectrum, one significant absorption peak at 1698 cm^−1^ was identified as a carboxyl group (CO-stretching vibration). From the highly resolved molecular mass of 299.2017 (M − H)^−^ and 323.1982 (M + Na)^+^, the molecular formula C_20_H_28_O_2_ could be deduced. According to the molecular formula, seven double bond equivalents were predicted for the structure, four of which relate to an aromatic ring, one to a carboxyl group and two to the aliphatic part of the molecule, which points to two aliphatic rings being attached to the aromatic ring. The ^1^H NMR spectra gave information on three aromatic-H (δ7.13, δ6.95, and δ6.85), which indicate a trisubstituted benzene ring (Table 1). In addition, 24 aliphatic-H were present, of which 12 belong to four CH_3_-groups (δ1.19, δ 1.22), and in addition two further aliphatic-H (δ2.85 and δ2.79) and further ten aliphatic protons (δ2.26–1.41) were indicated. The H from the carboxyl group was not visible. From COSY and HMBC spectra, the position of the isopropyl group at the aromatic ring was delineated. The ^13^C-NMR in connection with the DEPT and HSQC measurements gave information on the 20 C atoms (Table 1). One acidic carbonyl carbon (δ184.52), six aromatic carbons signals (δ147–124) (according to a phenyl group), and 13 aliphatic carbon signals (δ46.82–16.61) represented by four CH_3_, five CH_2_, two CH and two Cq (δ37.12 and δ 47.82) were detected. The oxygen is exclusively bound within a carboxyl group (δ184.52). According to the NMR analysis, the structure of F13, as shown in Figure 2, corresponds to dehydroabietic acid [13,14,15,16]. The connectivity of the carbon skeleton and the attached H-atoms and groups was determined by ^1^H/^1^H-COSY and HBMC spectra (Figure 3). The given absolute configuration is confirmed by the optical rotation [α_D_]^20^ = + 42 (*c* = 0.1 in methanol) and the CD maxima at 220 (ε 10,988) and 209 nm (ε 16.200).

### 2.5. Biological Activities

Compound **1** exhibited antibiotic activities only against the Gram-positive test strains. *Escherichia coli* and *Candida glabrata* were not inhibited. IC_50_ values against *Bacillus subtilis* and *Staphylococcus lentus* were 70.5 µM (±2.9) and 57.0 µM (±3.3), respectively. These values compare to IC_50_ values of chloramphenicol, of 1.45 µM (±0.13) and 2.13 µM (±0.11), respectively.

### 2.6. Genetic Approval of Secondary Metabolite Biosynthetic Pathways

PCR amplification for genes of established biosynthetic pathways for secondary metabolite production were performed using established primer systems for polyketides (*pks* I and *pks* II) non-ribosomal peptides (*nrps*), and phenazines (*phz*E fragments). None of these biosynthetic pathways could be detected by these PCR amplifications. However, searching with antiSMASH [17] in the genome of *Allochromatium vinosum* DSM 180^T^ demonstrated the presence of terpene biosynthesis gene clusters. In the established genome sequence of *Allochromatium vinosum* MT86, all steps of the biosynthesis of geranyl-PP and geranyl-geranyl-PP were present (data not shown), although indication for the reactions to diterpenoid biosynthesis, in particular abietic acid, could not be demonstrated. This failure could, however, be based on the lack of sufficient information in the genomic databases in regard to the diterpenoid biosynthesis pathway and may not exclude the presence of these biosynthetic steps. Therefore, proof for the biosynthesis of copalyl-PP and abietic acid by *Ach. vinosum* has to await future investigations.

## 3. Discussion

Dehydroabietic acid and abietic acid are metabolites known from plants and fungi and are characteristically produced by conifer trees. The compounds have quite recently also been identified in a variety of cyanobacteria and it was suggested that the evolutionary roots for their biosynthesis are indeed within the cyanobacteria, as the biosynthesis of abietadiene (a precursor of abietic acid) in the plants is located in the chloroplast organelles that are considered to be of endosymbiotic origin from cyanobacterial ancestors [13]. The only known terpene biosynthesis pathway in cyanobacteria, the methylerythrol pathway using pyruvate and glyceraldehyde-3-phosphate as substrates was supposed by theses authors to be involved in the synthesis of these two diterpenoids.

This is the first report of dehydroabietic acid from a proteobacterium, an anoxygenic phototrophic bacterium, produced under proper growth conditions and excreted into the culture medium. Although basic routes of the terpene biosynthetic pathway are common to all anoxygenic phototrophic bacteria, abietic acid and dehydroabietic acid have so far not been identified. Geranylgeranyl-PP is a central intermediate in the basic biosynthetic routes for carotenoid biosynthesis common to all phototrophic purple bacteria and also established in the genome of *Ach. vinosum* MT86. It is a precursor of phytoene and a diverse array of different carotenoids produced in these bacteria [18] as well as for the phytoyl moiety attached to the bacteriochlorophyll molecules of most phototrophic purple bacteria. In addition, hopanoid triterpenes produced by a number of anoxygenic phototrophic purple bacteria have geranylgeranyl pyrophosphate as precursor [19,20].

Dehydroabietic acid exhibits some interesting bioactivities including moderate antibiotic activities against *Bacillus subtilis* and *Staphylococcus lentus*, as found in the present study and also cytotoxic activities as demonstrated for a derivative of dehydroabietic acid recently [21]. Our study showed that the exploration of anoxygenic phototrophic purple bacteria for the production of bioactive compounds should be taken into consideration for biodiscovery.

## 4. Materials and Methods 

### 4.1. Isolation and Identification of Strain MT86

Strain MT86 was isolated from a sandy rock pool near to Trivendrum (Kerals, southwest coast of India) and pure cultures were obtained according to standard procedures [22]. Identification as a strain of *Allochromatium vinosum* was achieved using 16S rRNA gene sequences according to Gärtner et al. [23]. The 16S rRNA gene sequence (1353 nucleotides) was deposited in GenBank with the accession number MH678637.

### 4.2. Cultivation and Preparation of Cell Extracts

The strain was grown in Pfennigs medium amended with 1% thiosulfate and 1% acetate-solution (NH_4_-acetate/Mg-acetate, 2.5 g each/100 mL) using 1 L screw capped bottles. Well grown cultures were centrifuged to separate cell biomass and liquid supernatant. The supernatant was extracted with ethyl acetate (1:1 by volume); phase separation was allowed in a separatory funnel and the upper ethyl acetate phase was filtered (Whatman 595 ½) to remove solid impurities and water. The extract was concentrated to dryness in a rotary evaporator and resolved in a small volume of methanol. For control experiments, uninoculated medium was extracted directly by ethyl acetate without centrifugation steps and the extract treated the same way. All extracts were finally dissolved in methanol and filtered through 0.2 μm PTFE syringe filter before applying to analytical and semi-preparative HPLC.

### 4.3. Analytical HPLC-DAD/MS

The crude extract was re-dissolved in methanol to a concentration of 1.0 mg/mL and subjected to HPLC-DAD/MS using a VWR Hitachi Elite LaChrom system Elite (VWR, Darmstadt, Germany) with an L-2450 diode array detector, an L-2130 pump, and an L-2200 autosampler. This HPLC system was coupled to an ESI-ion trap detector with positive ionization (Esquire 4000, Bruker Daltonics, Faellanden Switzerland) for mass detection. A Onyx Monolithic reversed phase C18 column (100 mm × 3.00 mm; Phenomenex, Torrance, CA, USA) with solvent A (0.1% formic acid in 100% acetonitrile) and solvent B (0.1% formic acid in 100% Milli-Q water) was applied. The gradient parameters were: 0 min 95% A, 4 min 40% A, 6 min 0% A; flow: 2 mL/min).

### 4.4. Semi-Preparative HPLC

Semi-preparative HPLC was performed using a VWR Hitachi Elite LaChrom Elite HPLC system (VWR, Darmstadt, Germany). This system consisted of a C18 column (Phenomenex Gemini, 110 A AXIA, 100 mm × 50.00 mm) and a polar column (Phenomenex Synergi, 4 µL Polar-RP 80A, 250 mm × 21.20 mm) (Phenomenex, Torrance, CA, USA), an L-2200 autosampler (VWR, Darmstadt, Germany), a Foxy Jr. fraction collector (Teledyne Isco, Lincoln, NE, USA), an L-7150 pump, and an L-2450 diode array detector (VWR, Darmstadt, Germany). Solvents A and B were used with the following gradient: start 5 mL/min with 90% solvent A, after 0.5 min 10 mL/min 80% solvent A, after 26 min 15 mL/min 100% solvent A.

### 4.5. NMR Analysis

^1^H NMR, ^13^C NMR spectra and 2D spectra were recorded on an Inova 600 (600 and 125 MHz, respectively) with tetramethylsilane (TMS) as the internal standard and methanol-d(4) (MeOD) and CD_2_Cl_2_, respectively, as solvents. 

### 4.6. ESI-MS

Measurements of high-resolution mass spectra were performed on a Bruker-Apex III-Q mass spectrometer (field strength 7 Tesla) (Bruker Daltonics, Faellanden, Switzerland) with positive and negative ion mode.

### 4.7. Antimicrobial Activity of Compound ***1***

The following test organisms were used: *Bacillus subtilis* DSM 347 and *Staphylococcus lentus* DSM 6672 as representatives of Gram-positive bacteria; *Escherichia coli* DSM 498 as a Gram-negative bacterium; and the yeast *Candida glabrata* DSM 6425. All test organisms were obtained from the German Culture Collection (DSMZ, Braunschweig, Germany). The assays were performed according to Schneemann et al. [24]. Chloramphenicol (10 µM) and nystatin (10 µM) were used as positive control for the bacteria and the yeast, respectively.

## Figures and Tables

**Table 1:** ^13^C NMR and ^1^H-NMR spectroscopic data of compound **1** in CD_2_Cl_2_ (δ_C_ und δ_H_ in ppm).

No. of C	δ_C_	C-Atom	H-Atom	δ_H_	Position
1	184.52	COOH	1		C-18
2	147.23	C			C-9
3	145.89	C			C-13
4	134.99	C			C-8
5	127.03	CH	1	6.85	C-14
6	124.28	CH	1	7.13	C-11
7	123.99	CH	1	6.95	C-12
8	47.82	C			C-4
9	45.22	CH	1	2.15	C-5
10	38.40	CH_2_	2	2.26/1.41	C-1
11	37.20	CH_2_	2	1.74/1.62	C-3
12	37.12	C			C-10
13	33.87	CH	1	2.79	C-15
14	30.39	CH_2_	2	2.85	C-7
15	25.23	CH_3_	3	1.19	C-20
16	24.16	CH_3_	3	1.20	C-16
17	24.16	CH_3_	3	1.20	C-17
18	22.05	CH_2_	2	1.80/1.50	C-6
19	18.97	CH_2_	2	1.74/1.66	C-2
20	16.61	CH_3_	3	1.22	C-19
Total		20 C	28 H		
